# Editorial

**DOI:** 10.3897/zookeys.597.8618

**Published:** 2016-06-09

**Authors:** Michael Schmitt

**Affiliations:** 1 Ernst-Moritz-Arndt-Universitaet, Greifswald, Germany

The present volume will be the last one bearing the names of us three editors on the cover. Jorge Santiago-Blay has decided to step down from the board of editors (for family reasons), and Pierre Jolivet had done so already last year and returns only to bid farewell to Jorge.

Our co-operative editorship started in 2001, when Pierre Jolivet requisitioned Jorge and me when he planned to edit another volume on leaf beetles. This book grew enormously until the publisher compelled us to terminate further acceptance of contributions. Finally, even two chapters had to appear on a CD ROM that was inserted in the book. These chapters were one on leaf-mining chrysomelids and the extensive treatise on the subfamily Aulacoscelidinae/Aulacoscelinae, both by Jorge Santiago-Blay. “The green book” – *New Developments in the Biology of the Chrysomelidae* – contains 62 chapters by 111 authors from 27 countries. Jorge had been responsible for the majority of the manuscripts, which meant he had to find reviewers, correspond (and often discuss) with the authors, check the English, decide on final acceptance, and proofread. Thus, the biggest and heaviest book on leaf beetles depended for the most part on Jorge’s editorial efforts.

We could present the “Green Book” to the attendants of the Sixth International Symposium on the Chrysomelidae in May, 2004 in Bonn (Germany). At the same time, Pierre had already negotiated with Brill publishers about the launch of a new series, shortly after baptised *Research on Chrysomelidae*. As the co-operation of the three of us ran so harmoniously and productively, Pierre invited again Jorge and me to edit this series jointly. The first two volumes of this series appeared with Brill publishers (Leiden – Boston). Since the selling numbers did not meet the expectations of the publishers, they decided to drop the series after these two volumes. Again it was Pierre who found a solution: During the 9^th^ European Congress of Entomology, held in Budapest (Hungary) in 2010, he agreed with Lyubomir Penev from Pensoft publishers that *Research on Chrysomelidae* should be published as special issues of ZooKeys, again co-edited by the three of us. The present volume is the fourth, but certainly not the last, published by Pensoft. Although the pullout of Pierre Jolivet and Jorge Santiago-Blay marks a crucial cut in the history of *Research on Chrysomelidae*, I understand the reasons of their decision to step down. I hope and wish that the series will prosper and remain accepted as a forum of leaf beetle research by the community of Chrysomelidae enthusiasts all over the world. The next volume of *Research on Chrysomelidae* will contain the proceedings of the 9^th^ International Symposium on Chrysomelidae and will be co-edited by Caroline Chaboo (University of Kansas, Lawrence, Kansas, USA) and myself.

I thank Jorge Santiago-Blay from the bottom of my heart for his tireless engagement in fostering leaf beetle research and his friendship, and wish him All the Best for whatever he may entertain in the future.

Michael Schmitt (Greifswald, Germany)

